# Morphology and Mechanical Properties of 3D Printed Wood Fiber/Polylactic Acid Composite Parts Using Fused Deposition Modeling (FDM): The Effects of Printing Speed

**DOI:** 10.3390/polym12061334

**Published:** 2020-06-11

**Authors:** Teng-Chun Yang, Chin-Hao Yeh

**Affiliations:** Department of Forestry, National Chung Hsing University, Taichung 402, Taiwan; harrison19960219@gmail.com

**Keywords:** wood fiber, polylactic acid (PLA), fused deposition modeling (FDM), printing speed, morphology, mechanical properties

## Abstract

In this study, a wood fiber/polylactic acid composite (WPC) filament was used as feedstock to print the WPC part by means of fused deposition modeling (FDM). The morphology and mechanical properties of WPC parts printed at different speeds (30, 50, and 70 mm/s) were determined. The results show that the density of the printed WPC part increased as the printing speed decreased, while its surface color became darker than that of parts printed at a high speed. The printing time decreased with an increasing printing speed; however, there was a small difference in the time saving percentage without regard to the dimensions of the printed WPC part at a given printing speed. Additionally, the tensile and flexural properties of the printed WPC part were not significantly influenced by the printing speed, whereas the compressive strength and modulus of the FDM-printed part significantly decreased by 34.3% and 14.6%, respectively, when the printing speed was increased from 30 to 70 mm/s. Furthermore, scanning electronic microscopy (SEM) illustrated that the FDM process at a high printing speed produced an uneven surface of the part with a narrower width of printed layers, and pull-outs of wood fibers were more often observed on the fracture surface of the tensile sample. These results show that FDM manufacturing at different printing speeds has a substantial effect on the surface color, surface roughness, density, and compressive properties of the FDM-printed WPC part.

## 1. Introduction

In recent years, additive manufacturing (AM), commonly referred to as 3D printing, has seen increased use as a low-cost and versatile technology for rapid prototyping. In comparison to traditional subtractive manufacturing, the AM technology creates 3D objects by a layer-by-layer process of joining materials to avoid unnecessary waste while obtaining high geometric accuracy [1,2]. This technology enables the automatic fabrication of designed objects with a complex shape by the assembly of individual layers and is unhindered by design complexity [3,4]. Commercially available AM methods include stereolithography (SLA), laminated object manufacturing (LOM), selective laser sintering (SLS), inkjet printing (IP), and fused deposition modeling (FDM). Among these methods, FDM is one of the most popular and widely used type of desktop 3D printer since it has several advantages: a low manufacturing cost (relatively low-cost materials and a low initial investment), the ability to be operated in laboratory or office environments, the reduction of material waste, and the ability to fabricate products with different colors or materials [5].

Conventional or biodegradable thermoplastic polymer materials such as polylactic acid (PLA), polypropylene (PP), and acrylonitrile butadiene styrene (ABS) can be processed through the FDM method to form solid parts with a 3D geometry by assembling successive layers [5,6,7,8,9,10,11]. Due to various selections of materials, the 3D printing of polymeric products has been explored in various applications, such as aeronautics, civil engineering, art, and medical fields [12,13,14,15]. Among these pure materials, PLA is a biodegradable polymer with the potential to replace petroleum-based plastics due to its biodegradability and exceptional mechanical properties. However, on the downside, it is well known that PLA has a low thermal resistance and is brittle, restricting its application in a wide variety of 3D printed polymers as load-bearing and functional parts. To overcome these drawbacks, the addition of fibers into a polymeric matrix allows for the fabrication of a composite with exceptional mechanical properties and excellent functionality. Several previous studies have reported that natural fibers can be incorporated into PLA as reinforcements to improve its properties due to their numerous positive characteristics: renewability, biodegradability, low density, low cost, and advantageous specific properties [16,17,18]. Faruk et al. [19] explained that natural fiber-reinforced PLA composites, commonly known as biobased composites, have received much attention owing to their specific mechanical properties, controlled environmental footprint, and suitable end-of-life management. Therefore, the use of natural fiber/PLA composite filaments in FDM methods has become increasingly attractive due to their low environmental impact and material cost.

In the FDM printing process, a control model supports the working principle of the entire system, which combines various parameters, namely structural, processing, and extruder-related parameters [20]. Previous studies have reported that process parameters significantly affect the quality and mechanical properties of FDM-printed parts, such as the printing orientation, layer thickness, feed rate, infill density and pattern, printing speed, and extrusion temperature [7,8,9,11,21,22,23,24,25,26,27]. Several studies [8,9,22] have shown that the printing orientation significantly changes the structures of printed parts, resulting in a variation of their mechanical properties. Furthermore, aspects of printed parts are influenced by the printing orientation, such as dimensional accuracy, surface roughness, and printing time and cost [9]. Chacón et al. [7] indicated that a decrease in the tensile and flexural strengths of upright-printed PLA parts was observed as the feed rate increased. Chacón et al. [7] and Tymrak et al. [8] showed that the tensile strength of the FDM-printed PLA product increases and its flexural strength decreases with a decrease in the layer thickness. Tsouknidas et al. [11] explored the tensile properties and impact behavior of FDM-printed PLA parts with various infill patterns and densities and printing speeds. Additionally, studies concerning the mechanical properties of printed carbon fiber/plastic composites and wood fiber/PLA composites (WPCs) with different extrusion temperatures have been performed by Yang [21] and Ning et al. [23]. Generally, the extrusion temperature for PLA composites is set above 200 °C in the FDM printing process. The heating temperature significantly affects the color, physical, and mechanical properties of wood or composites with heat-treated wood fibers due to the variation in the chemical composition of lignocellulosics when the heating temperature is above 180 °C, such as carbohydrate depolymerization, hemicellulose degradation, and the crosslinking of lignin-carbohydrate [28,29,30,31,32]. In the FDM printing process, the rheological behavior of the filament, crystallinity and deformation of the layer, and internal bonding between layers are influenced by temperature, which indirectly affects the FDM printability and macromechanical properties of the printed parts [21,23,33,34,35,36,37]. Hence, temperature is one of the main effective process parameters in the FDM method. Except for the extrusion temperature, the increase in the feed rate can reduce the heating time of the melt filaments through the heater in the nozzle, or a sufficiently fast printing speed can minimize the holding time for the layers at dangerous temperatures to prevent thermal degradation of the lignocellulosic fibers in the filament [5,20]. These essential process parameters work together to control the speed of an FDM system. As described above, there is a certain interactive effect between the temperature and the printing speed in the FDM printing process of the WPC part. However, to the best of our knowledge, the effect of printing speed on the characteristics of the FDM-printed WPC part has not yet been systematically studied. Hence, the present study aims to investigate the morphology and mechanical properties of WPC parts using FDM printing at different printing speeds.

## 2. Materials and Methods

### 2.1. Materials

Commercial WPC filament (40 wt % wood fiber and 60 wt % PLA) having a diameter of 1.75 mm was purchased from Formfutura BV (Gelderland, The Netherlands). The density of the WPC filament was 1.20 g/cm^3^, its melting point was 145 ± 10 °C, and its melt flow index was 4.5 g/10 min.

### 2.2. Preparation of FDM-Printed WPC Parts

**Figure 1:**
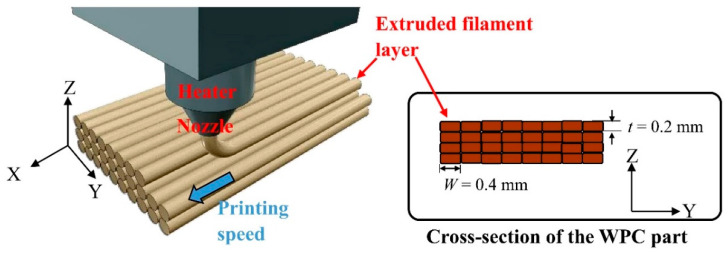
Schematic of a customized FDM system and the cross-section of the WPC part printed with extruded filament layers.

All WPC parts were fabricated with a 0.4 mm nozzle using a customized FDM printer (Creator Pro, Flashforge Corp., Zhejiang, China). The process parameters were set in Flashprint software combined with Slicer software to control the printer. As depicted in Figure 1, the extruded filament layers were assembled by layer-by-layer movements of the nozzle along the printing axis (*X*-axis) and without a contour. The structural part of the WPC sample was fabricated by extruded filament layers with a printing layer thickness (*t*) and width (*W*) of 0.2 mm and 0.4 mm, respectively. Three printing speeds (30, 50, and 70 mm/s) were used to print the samples, which were designated WPC_30_, WPC_50_, and WPC_70_, respectively. During printing, the temperature of the build platform was 50 °C, and the extrusion temperature was 210 °C. Additionally, the printing times of the printed parts were recorded, and the time saving percentage (TSP) was calculated as compared to the time required for WPC_30_.

### 2.3. Determining Properties of the Printed Parts

**Figure 2:**
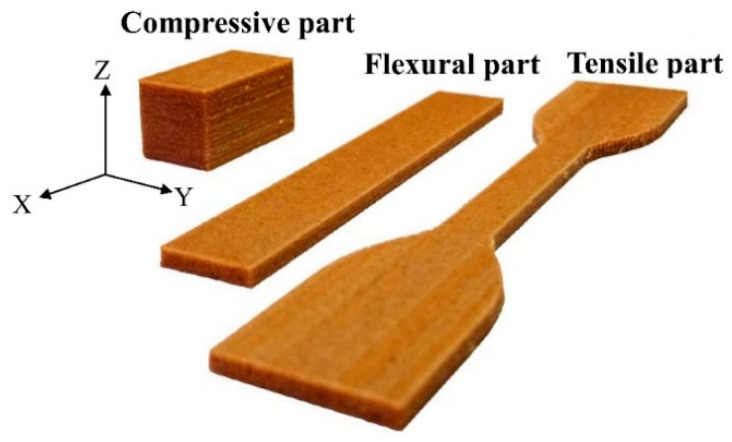
Appearances of the FDM-printed WPC parts for the tensile test, flexural test, and compressive test.

**Figure 3:**
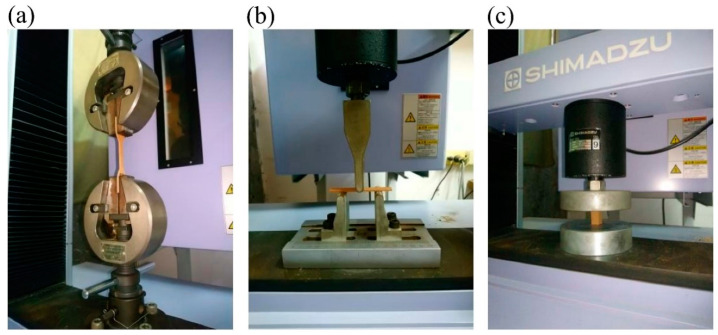
Setups of the tensile test (**a**), flexural test (**b**), and compressive test (**c**).

The tensile properties, flexural properties, and compressive properties of the printed samples were determined according to the ASTM standards. The tensile strength (TS) and tensile modulus (TM) values were evaluated from samples with dumbbell shape (type IV) at a span of 65 mm and a tensile speed of 5 mm/min (ASTM D638). The modulus of rupture (MOR) and modulus of elasticity (MOE) values were assessed from samples with dimensions of 80 mm × 12 mm × 3 mm using a three-point flexural test at a span of 48 mm and a crosshead speed of 1.28 mm/min in accordance with ASTM D790. According to the ASTM D695, the compressive strength (CS) and compressive modulus (CM) values were obtained at a crosshead speed of 1.3 mm/min (sample size: 12 mm × 12 mm × 24 mm). The densities of the tensile and compressive samples were determined. The appearances of the tensile, flexural, and compressive parts and various test setups are shown in Figure 2 and Figure 3. All samples were conditioned at 20 °C and 65% relative humidity (RH) for 14 days before testing.

### 2.4. Differential Scanning Calorimetry (DSC)

Heat flow was recorded using a PerkinElmer DSC-6 (Beaconsfield, UK). Approximately 3 mg of the filament sample was weighed in DSC aluminum pans with lids and operated under a continuous flux of nitrogen at a flow rate of 20 mL/min. To eliminate the thermal and mechanical history and ensure a nuclei-free melt, the first step was to heat the sample to 200 °C at a constant heating rate of 10 °C/min, and this temperature was held for 5 min. Then, the sample was cooled to 50 °C at a constant cooling rate of 10 °C/min, followed by a second heating to 200 °C with 10 °C/min.

### 2.5. Morphology of the Printed Parts

SEM micrographs for surface appearances of the parts and fracture cross-sectional surfaces of the tensile samples were obtained using a JEOL JSM6330F SEM (Tokyo, Japan) with a field emission gun and an acceleration voltage of 3.0 kV. All samples were dried and sputtered with platinum before testing.

### 2.6. Surface Color

The CIE L*a*b* colors on the surface of the printed WPC part were measured by a spectrophotometer (Minolta CM-3600d, Japan) under a D65 light source. The color difference (ΔE*) is calculated by the following equation:

ΔE* = [(ΔL*)^2^ + (Δa*)^2^ + (Δb*)^2^]^1/2^ (1)

where L* is the value of the white/black axis, a* is the value of the red/green axis, and b* is the value on the yellow/blue axis.

### 2.7. Statistical Analysis

All the characteristic results for WPC parts were expressed as the mean ± the standard deviation (SD). The significance of the differences was calculated using Scheffe’s test; *p* < 0.05 was considered to be significant.

**Figure 4:**
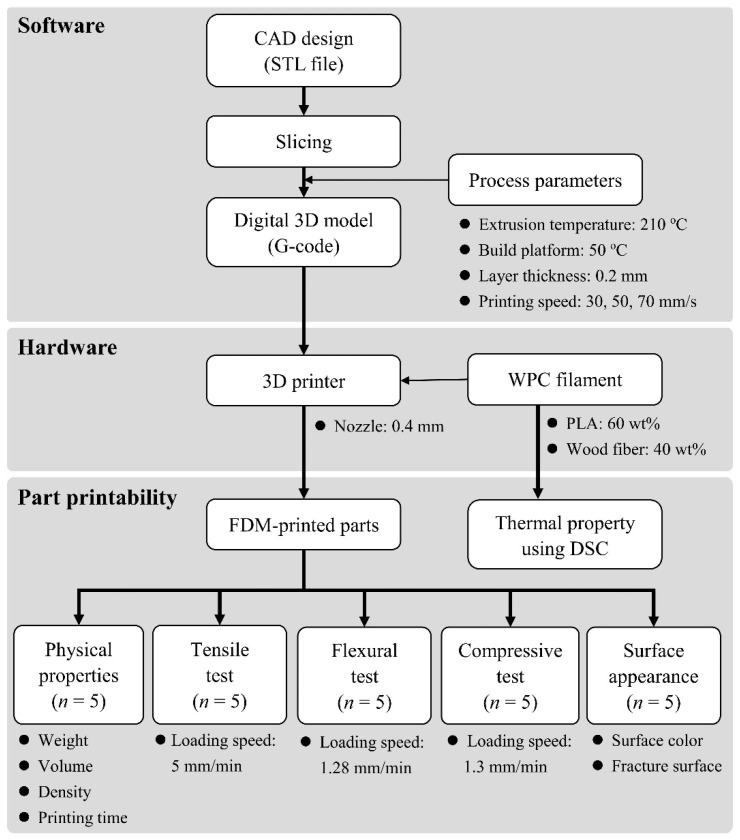
Work plan of the present study.

To a better understanding, Figure 4 schematically presents the work plan of the present study, from the FDM printing to the printability and physicomechanical properties of the printed parts.

## 3. Results and Discussion

### 3.1. DSC Measurement

**Figure 5:**
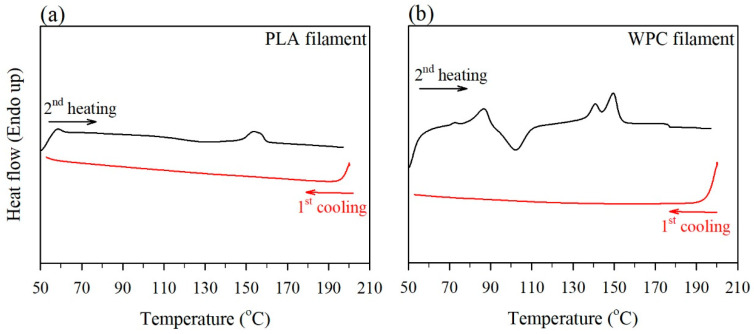
DSC thermograms of the PLA filament (**a**) and WPC filament (**b**) at the the first cooling process (10 °C/min) and the subseguent second heating process (10 °C/min).

In the FDM printing process, the WPC filament was melted in a heater at 210 °C and cooled on a build platform at 50 °C. To simulate this situation and to understand the properties of the FDM-printed part, the effect of wood fiber on the thermal behavior of the PLA matrix in the WPC filament was determined by DSC measurments. Generally, the properties of polymeric products are influenced by the formation of crystalline structures and crystallinity indices upon heating and cooling processes. Figure 5 shows the heat flow of the melted PLA filament (neat PLA) and WPC filament at the first cooling scan and at the second heating scan after cooling. It can be seen that the glass transition temperature of PLA was approximately 60 °C for all samples during the second heating process. When wood fibers were added into PLA (Figure 5b), the cold crystallization exotherm was observed in the range of 90–110 °C upon the second heating. Then, two peak melting temperatures were obtained at 140 and 147 °C from the DSC curve of the WPC filament. The former peak is associated with the melting of crystals of various sizes formed by the conformational purity and chain alignment of the PLA matrix [38]. The latter peak at the higher temperature is attributed to the fusion of perfect crystals produced through the cold crystallization process [38]. Additionally, the exothermic melting crystallization of the PLA matrix was not detected for all samples upon cooling at 10 °C/min (Figure 5). According to previous studies [39,40,41,42], the crystallization of pure PLA was not observed upon cooling due to its low crystallization rate compared to other semicrystalline polymers. Several studies have also reported that the presence of lignocellulosic reinforcement can act as a nucleating agent to increase the crystallization rate of the polymeric matrix, indicating that crystallization exotherm was observed upon cooling [39,41,43,44,45,46]. However, in this study, the results implied that the PLA matrix was not crystallized in the WPC parts during FDM printing because its crystallization rate was too slow to reach crystal completion.

### 3.2. Surface Color

**Figure 6:**
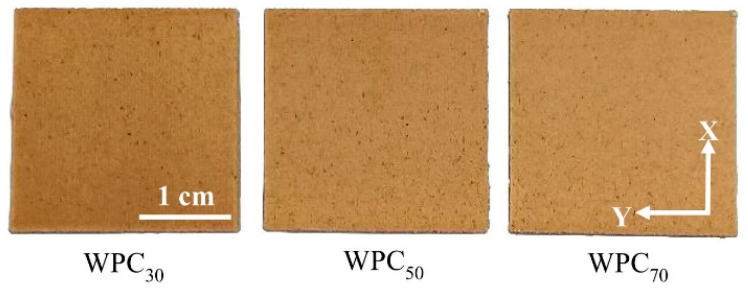
Surface appearances of FDM-printed WPC parts at various printing speeds.

Color measurements were used to investigate the thermal degradation degree of the wood fibers in the FDM-printed WPC part in this study. As shown in Figure 6, the surface color of WPC_70_ was slightly brown, and a darker brown color was observed when the printing speed was 30 mm/s (WPC_30_). These results demonstrated that the surface color trait of the printed sample was influenced by the printing speed and that the color of the printed WPC part became darker as the printing speed decreased. This behavior is related to the fact that the movement of the nozzle at a low printing speed is slow, resulting in more heat treatment of the wood fibers, leading to a darker WPC part. The color variation of heat-treated wood is related to changes in polysaccharides, such as the formation of oxidation products; changes in extractives; and the formation of products from hemicellulose decomposition [47,48]. Table 1 presents the effect of the printing speed on the color parameters of the FDM-printed WPC part. The L* value significantly decreased from 57.1 for WPC_70_ to 54.2 for WPC_30_, indicating that the L* value decreased as the printing speed decreased. Additionally, the a* and b* values were not influenced by the printing speed in the ranges of 15.4–15.8 and 22.1–22.4, respectively. On the other hand, compared to WPC_30_, the color difference (ΔE*) increased to 3.0 when the printing speed reached 70 mm/s (WPC_70_). Previous studies have reported that a decrease in L* and an increase in ΔE* of the heat-treated wood are caused by hemicellulose degradation [49,50]. This result confirms that the printing speed affects the color of the wood fibers in the WPC part.

### 3.3. Density, Printing Time, and Surface Morphology

**Figure 7:**
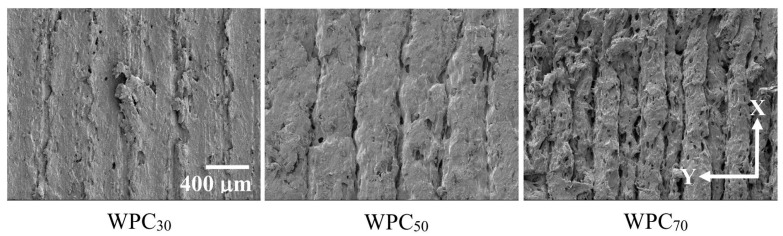
SEM micrographs of the surface morphologies of the WPC parts printed at various printing speeds.

**Figure 8:**
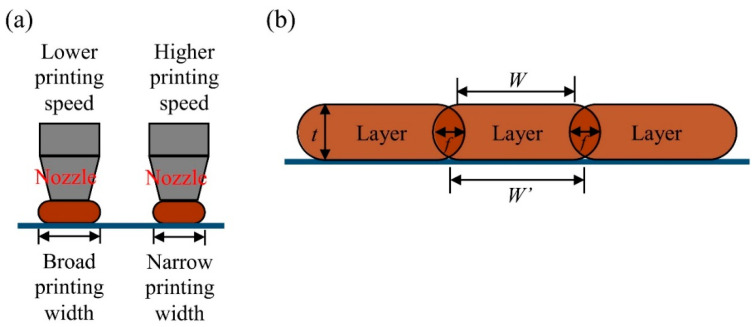
Schematics of the influence of printing speed on the printing width of the layer (**a**) and the adjacent layer with the overlapped offset (**b**).

In this section, the flexural WPC part (WPC_f_) with a smaller dimension was compared with the compressive part (WPC_c_) with a larger dimension to understand the effects of the dimension and printing speed on the density and printing time. As shown in Table 2 and Table 3, when the printing speed increased from 30 to 70 mm/s, the weight (*w*) of the printed WPC_f_ and WPC_c_ significantly decreased from 2.55 to 2.40 g and from 3.90 to 3.44 g, respectively. This result exhibited that the nozzle movement at a low printing speed is slow, resulting in depositing more materials on the same path to increase the weight of the printed part. However, the volume (*V*) was not significantly different among individual samples in the ranges of 2.39–2.73 cm^3^ for WPC_f_ and 3.58–3.65 cm^3^ for WPC_c_. Hence, since the *w* per volume decreased with an increase in printing speed, the WPC part printed at 70 mm/s showed the lowest density among individual samples (1.00 g/cm^3^ for WPC_f_ and 0.96 g/cm^3^ for WPC_c_). These results indicated that the density of the WPC part decreased with an increasing printing speed. Additionally, compared to the density of the individual WPC_30_, the density decreased by 5.9% for WPC_f70_ and by 10.3% for WPC_c70_. These results implied that the degree of reduction in the density of a WPC part with a larger size (e.g., WPC_c_) was high at a high printing speed. On the other hand, the printing times were 39.4, 26.6, and 22.6 min for WPC_f30_, WPC_f50_, and WPC_f70_, respectively. Comparted to WPC_f30_, the time saving percentage (TSP) was 32.5% for WPC _f50_ and 42.6% for WPC _f70_. Similarly, for the compressive WPC part, the printing time decreased from 61.0 min (WPC_c30_) to 36.4 min (WPC_c70_). In addition, the TSP values were 30.5% for WPC_c50_ and 40.3% for WPC_c70_. According to these results, it can be concluded that the printing time decreased with an increasing printing speed; however, the TSP value at a given printing speed for the WPC part may be slightly different regardless of the dimension of the printed WPC part. Figure 7 presents SEM micrographs showing the surface morphologies of the WPC parts at various printing speeds. The layer width of the WPC_30_ was broader than the expected printing width of 0.4 mm. When the printing speed reached 70 mm/s, the printed layer width was less than 0.4 mm. Therefore, the printed layer width decreased and the roughness of the surface of the part increased with an increasing printing speed. Additionally, this phenomenon further confirmed that the density of the FDM-printed WPC part decreased as the printing speed increased. Carneiro et al. [5] stated that an increase in printing speed leads to a decrease in the layer width at a given thickness for the layer. Figure 8a shows the effect of printing speed on the printing width of the layer. The adjacent layer with the overlapped offset (*f*) is illustrated in Figure 8b according to the geometry model presented by several studies [24,25,51,52]. The actual width (*W’*) with offset (*f*) is determined using the following equations:

*W’* = *W* + *t* − *f* (2)

*f* = (*π* − 4)*t*/4 (3)

where *t* is the layer thickness. When the printing speed is lower, excess deposited layers and a higher *f* value can be caused by very closed adjacent layers. In contrast, for the part printed at a higher printing speed, a lower *f* value due to a nonexistent or minimal bonding interface between adjacent layers weakened the structural integrity of the printed part. As described above, in the present study, the results confirmed that the *f* value of WPC_30_ was higher than that of WPC_70_.

### 3.4. Fracture Morphology and Mechanical Properties

The mechanical properties of FDM-printed parts at various printing speeds are listed in Table 4. All of the WPC parts showed tensile strength (TS) and tensile modulus (TM) values in the ranges of 19.2–19.8 MPa and 1650–1731 MPa, respectively, indicating that they were not influenced by the printing speed. Similarly, there were no significant differences in the modulus of rupture (MOR) and modulus of elasticity (MOE) values among all samples with various printing speeds (32.3–34.0 MPa for MOR and 1575–1680 MPa for MOE). These results indicated that the tensile and flexural properties of the FDM-printed WPC part were independent of printing speed. As described in the previous section, the wood fibers can be heated to cause thermal modifications during the FDM printing process since the WPC filament is fed through the heater with temperature of 210 °C. Therefore, the lack of a significant difference in the tensile and flexural performances of the printed parts among individual samples with various printing speeds may be attributed to the interplay of two scenarios: (i) good fiber/PLA bonding interactions due to the improvement in the compatibility of wood fibers and (ii) the thermal degradation of wood fibers. In the former case, wood fibers are modified to be hydrophobic by a heat-induced physical treatment, increasing the fiber/polymer bonding adhesion so that they stick together [53,54]. Thus, the stress can be smoothly transmitted between two faces [55,56], resulting in an increase in the tensile and flexural properties of the WPC parts. In the latter case, the acidic products formed from the thermal degradation of wood fibers cause the cleavage of C-C and C-O linkages at the intrapolymer level and the depolymerization and shortening of the cellulose polymer in fibers [32,57,58], resulting in a loss of the tensile and flexural properties of the printed WPC part. Taking these scenarios into account, no significant changes in the tensile and flexural properties of the WPC parts could be attributed to a counterbalance of the increased compatibility of the fiber/PLA interfacial surface and the thermal decomposition of wood fibers. Similar results were reported in the work by Yang et al. [54].

**Figure 9:**
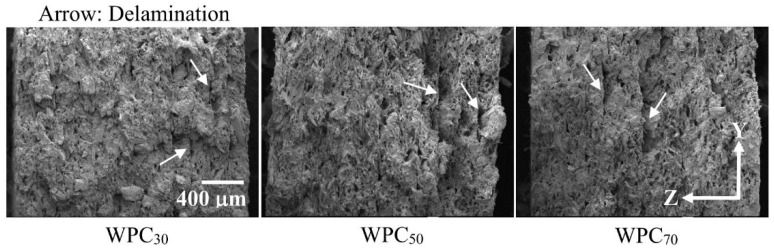
SEM micrographs of the fracture cross-sectional surfaces of WPC parts printed at various printing speeds.

**Figure 10:**
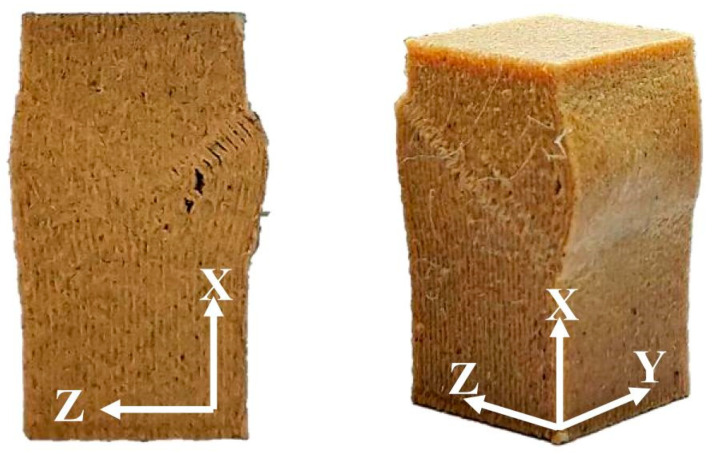
Compressive failure appearances of the FDM-printed WPC part.

Figure 9 provides SEM micrographs of the fracture cross-sectional surfaces of the WPC parts at various printing speeds after tensile testing. In the case of WPC_30_, few fiber pull-outs and fiber breakages occurred on the fracture surface. This phenomenon may be related to the thermal degradation of the wood fibers and good fiber/PLA bonding interactions since the wood fibers were more heat treated at the lower printing speed. When the printing speed increased to 70 mm/s (WPC_70_), pull-outs of wood fibers were clearly observed more often compared to the part printed at a lower printing speed. This phenomenon occurs with most wood fibers, which have poor adhesion with the PLA matrix. Furthermore, WPC_30_ exhibited the highest CS (31.8 MPa) and CM (852 MPa) values among all the samples. As the printing speed increased to 70 mm/s, the CS and CM values of the part (WPC_70_) significantly decreased by 34.3% and 14.6%, respectively, comparted to those of the WPC_30_. When the printed part was subjected to longitudinal compression along the printing orientation (*X*-axis), the internal bonding strength between adjacent layers mostly affected the compressive load-carrying capacity of the WPC part [21]. Therefore, the results indicated that the WPC_70_ had the lowest bonding strength between layer interfaces due to minimal bonding between adjacent layers. In contrast, when the printing speed decreased to 30 mm/s, more overlapping adjacent layers resulted in better bonding and thus better compressive performance. As shown in Figure 10, interface debonding between adjacent layers in the damage evolution of all the samples was observed as a result of the kinking and buckling of the filament layers. Additionally, the transverse deformation of the compressed WPC part mainly occurred along the *Z*-axis rather than along the *Y*-axis (Figure 8). This behavior is attributed to the fact that the interlayer bonding in the *Z*-axis is poorer than that in the *Y*-axis. From the fracture patterns in the broken cross-sections (Figure 9), delamination was observed between two layers in the XY-plane due to weak bonding in the *Z*-axis at various printing speeds. As mentioned above, these results summarize that FDM printing at a high printing speed can decrease the compressive performance of the WPC part, whereas the tensile and flexural properties of the printed samples are independent of the printing speed.

## 4. Conclusions

The present study investigated the morphology and mechanical properties of WPC parts prepared by FDM printing at different printing speeds in the range of 30–70 mm/s. Compared to the WPC_30_ sample, a decrease in L* and an increase in ΔE* on the surface of printed samples were observed as the printing speed increased. The density of the WPC part decreased with an increasing printing speed, and the degree of reduction in the density of a large WPC part was high at a high printing speed. Additionally, the printing time decreased with an increasing printing speed; however, the difference in the time saving percentage at a given printing speed was small regardless of the dimension of the printed part. On the other hand, there was no significant difference in the tensile and flexural properties among all the printed samples. Moreover, the compressive properties of the printed samples decreased with an increase in printing speed due to the reduction in the bonding strength of the adjacent layers. In the SEM micrographs, a decrease in the printed layer width was observed to cause an uneven surface of the WPC part printed at a higher printing speed, and the fracture surface of the tensile sample showed pull-outs of wood fibers more often than the other samples. Accordingly, the performance of the FDM-printed WPC part is influenced by the printing speed. These results provide information on improving and optimizing the FDM-printed manufacturing process of the WPC part.

## Figures and Tables

**Table 1:** Surface color parameters of FDM-printed WPC parts at various printing speeds.

Code	Printing Speed (mm/s)	L*	a*	b*	ΔE*
WPC_30_	30	54.2 ± 0.7 ^c^	15.8 ± 0.5 ^a^	22.2 ± 0.2 ^a^	-
WPC_50_	50	55.7 ± 0.6 ^b^	15.7 ± 0.5 ^a^	22.1 ± 0.1 ^a^	1.6 ± 0.5 ^b^
WPC_70_	70	57.1 ± 0.7 ^a^	15.4 ± 0.2 ^a^	22.4 ± 0.3 ^a^	3.0 ± 0.7 ^a^

The values are the mean ± SD (*n* = 5). Different letters (a, b, and c) within a column indicate a significant difference at *p* < 0.05.

**Table 2:** Density and printing time of flexural WPC parts printed at various printing speeds.

Code	Printing Speed (mm/s)	*w* (g)	*V* (cm^3^)	Density (g/cm^3^)	Printing Time (min)	TSP (%)
WPC_f30_	30	2.55 ± 0.02 ^a^	2.41 ± 0.05 ^a^	1.06 ± 0.02 ^a^	39.4 ± 0.5 ^a^	-
WPC_f50_	50	2.47 ± 0.01 ^b^	2.41 ± 0.02 ^a^	1.02 ± 0.01 ^b^	26.6 ± 0.5 ^b^	32.5
WPC_f70_	70	2.40 ± 0.02 ^c^	2.39 ± 0.01 ^a^	1.00 ± 0.01 ^b^	22.6 ± 0.5 ^c^	42.6

The values are the mean ± SD (*n* = 5). Different letters (a, b, and c) within a column indicate a significant difference at *p* < 0.05.

**Table 3:** Density and printing time of the compressive WPC parts printed at various printing speeds.

Code	Printing Speed (mm/s)	*w* (g)	*V* (cm^3^)	Density (g/cm^3^)	Printing Time (min)	TSP (%)
WPC_c30_	30	3.90 ± 0.03 ^a^	3.65 ± 0.01 ^a^	1.07 ± 0.01 ^a^	61.0 ± 0.7 ^a^	-
WPC_c50_	50	3.74 ± 0.03 ^b^	3.63 ± 0.03 ^a^	1.03 ± 0.01 ^b^	42.4 ± 0.5 ^b^	30.5
WPC_c70_	70	3.44 ± 0.02 ^c^	3.58 ± 0.02 ^a^	0.96 ± 0.02 ^c^	36.4 ± 0.5 ^c^	40.3

The values are the mean ± SD (*n* = 5). Different letters (a, b, and c) within a column indicate a significant difference at *p* < 0.05.

**Table 4:** Mechanical properties of FDM-printed WPC parts at various printing speeds.

Code	Tensile Properties		Flexural Properties		Compressive Properties	
	TS (MPa)	TM (MPa)	MOR (MPa)	MOE (MPa)	CS (MPa)	CM (MPa)
WPC_30_	19.8 ± 0.8 ^a^	1731 ± 60 ^a^	34.0 ± 1.5 ^a^	1680 ± 78 ^a^	31.8 ± 0.6 ^a^	852 ± 8 ^a^
WPC_50_	19.2 ± 0.7 ^a^	1650 ± 60 ^a^	33.1 ± 0.5 ^a^	1620 ± 70 ^a^	27.7 ± 0.9 ^b^	825 ± 11 ^a^
WPC_70_	19.8 ± 0.3 ^a^	1682 ± 27 ^a^	32.3 ± 0.4 ^a^	1575 ± 34 ^a^	20.9 ± 1.8 ^c^	728 ± 28 ^b^

The values are the mean ± SD (*n* = 5). Different letters (a, b, and c) within a column indicate a significant difference at *p* < 0.05.
